# Indium Incorporation into Tungsten Disulfide Monolayers during Chemical Vapor Deposition Growth: Doping and Enhancement of Photoluminescence

**DOI:** 10.1021/acsomega.5c05880

**Published:** 2025-09-15

**Authors:** Neileth Stand, Cesar D. Mendoza, Fernando Lázaro Freire

**Affiliations:** † Pontifícia Universidade Católica do Rio de Janeiro, Departamento de Física, Rua Marquês de São Vicente, 225, Rio de Janeiro 22451-900, Brazil; ‡ Universidade do Estado do Rio de Janeiro, Departamento de Engenharia Elétrica, Rua São Francisco Xavier, 524, Rio de Janeiro 20550-900, Brazil

## Abstract

Tungsten disulfide (WS_2_) monolayers have attracted significant attention due to their remarkable electrical and optoelectronic properties, making them promising candidates for next-generation nanoelectronic and optoelectronic devices. In this study, we employed a multitechnique approach to investigate the effects of indium (In) atom incorporation into WS_2_ monolayers during chemical vapor deposition growth. Photoluminescence spectroscopy revealed up to a 4-fold increase in emission intensity. Raman spectroscopy showed that the crystalline structure remained intact despite the incorporation of In atoms. X-ray photoelectron spectroscopy (XPS) confirmed the presence of In atoms bonded to both tungsten (W) and sulfur (S). The observed shifts in peaks corresponding to W^4+^ and S^2–^ oxidation states—characteristic of WS_2_—along with the change in the valence band maximum (VBM) position, indicated a Fermi level shift toward the valence band, signifying p-type doping. Atomic force microscopy (AFM) and scanning electron microscopy (SEM) were used to analyze the topography and morphology of the samples. These findings provide a foundation for the controlled synthesis of In-doped WS_2_, facilitating targeted optoelectronic applications.

## Graphical abstract

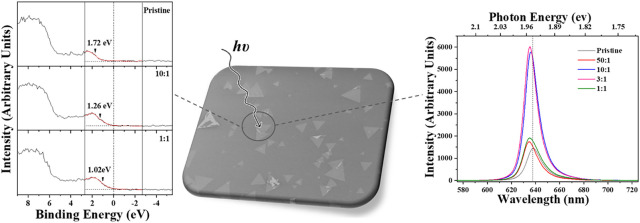

## 1 Introduction

Two-dimensional (2D) transition metal dichalcogenides (TMDs) feature a hexagonal structure in which transition metal atoms are sandwiched between two layers of chalcogenide atoms. Certain semiconducting TMDs, such as WS_2_, undergo an indirect-to-direct bandgap transition when a single, atomically thin layer is isolated from the bulk.^1^ WS_2_ is regarded as a promising multifunctional material for optoelectronic and electronic applications due to its strong luminescence, robust spin–orbit coupling, atomic-scale thickness, and lack of dangling bonds. TMD materials are primarily intended to be integrated into systems that overcome the scaling limits of current silicon technology.^2–5^

To achieve the applications mentioned earlier, it is essential to obtain the TMDs under controlled conditions that ensure consistent quality, size, and reproducibility compatible with industrial processes. The chemical vapor deposition (CVD) method is particularly suitable due to its low cost, simplicity, and versatility in controlling various synthesis parameters, making it more practical than mechanical exfoliation, which is less feasible on an industrial scale.^6^ Additionally, the properties of atomically thin semiconductor materials can be engineered to facilitate the construction of fundamental nanoelectronic components, such as the PN junction, thereby enabling the development of next-generation logic circuits. This approach requires strategies for controlling or intentionally modifying the concentrations of charge carriers.^7–9^ Building on processes developed for silicon, various studies have explored the controlled incorporation of defects, surface functionalization, and impurity introduction into the structure of the material. These modifications effectively alter carrier concentrations and influence other physicochemical properties.^10^ A recent review provides a detailed discussion of the potential applications of doped TMD monolayers.^11^

Despite achieving high-quality, large-area material production, intrinsic defects still exist during the preparation process. These defects are primarily caused by a high density of sulfur vacancies, which directly impact the quantum yield of photoluminescence, charge carrier concentration, and catalytic activity.^5^ In WS_2_, such defects create features such as deep donor energy levels, approximately 0.45 eV below the conduction band, intrinsic n-type doping, and an enhanced ability to amplify the effects of various bodies acting as nonradiative recombination centers. One of the most effective strategies to mitigate these native defects is to introduce heteroatomos into the materials, enabling p-type doping.^12^

Recent years have seen extensive research on substituting transition metal atoms or passivating sulfur vacancies to enable applications in electronics and optoelectronics.^10,13^ Substitutional doping—where impurity atoms replace metal cations with different valencies—effectively modulates carrier types and densities.^14–16^ The methods to achieve this doping vary and can be performed during or after material preparation (*in situ* or *ex-situ*). The first method is notable for its scalability, utilizing a mixture of various precursors—both solid and liquid—along with salts that facilitate material synthesis. The second involves techniques such as ion bombardment or plasma treatment, where the dosing mechanism plays a significant role.^17^

When TMDs are doped, their impact on the crystalline structure is significant because defects are introduced and the material properties are modified. In the context of developing optoelectronic devices and enhancing photoluminescence properties, achieving p-type doping is also a crucial aspect. Among various approaches, the incorporation of boron, gallium, and indium atoms into WS_2_ monolayers synthesized by chemical vapor deposition (CVD) has been investigated. Regarding indium doping, a first study was carried out by Ying Chen and collaborators. They optimized the precursor mass ratios (In_2_O_3_ and WO_3_), using NaCl salt as a catalyst, and observed an improvement in the photoluminescence of doped samples.^18^ However, their findings present some contradictions. Although a blue shift in the photoluminescence peak was observed in In-doped samples, the Raman peaks exhibited a redshift of 2 cm^–1^ compared to undoped WS_2_. These results do not clarify the type of doping achieved or whether strain has a significant influence on the outcomes. Additionally, the authors did not thoroughly analyze their X-ray photoelectron spectroscopy (XPS) data, instead considering the W4f core level region as a whole and overlooking the possible presence of different chemical environments. The presence of indium atoms at tungsten sites within the lattice was revealed through scanning transmission electron microscopy (STEM) images, and only highly doped samples demonstrated p-type behavior.^18^ In a recent study, the same group conducted a systematic investigation that combined both theoretical and experimental approaches.^3^ In this investigation, WS_2_ samples were doped with group IIIA atoms (B, Ga, and In). The authors claimed that the doped samples possess both giant photoluminescence quantum yield enhancement and effective carrier polarity modulation. Here, the photoluminescence increased for the doping atoms (B > Ga > In), which was consistent with observations in STEM images, where indium atoms preferentially substitute for W atoms. In contrast, gallium atoms both fill vacancies of S atoms and substitute W atoms, and boron atoms were more likely to fill vacancies of S atoms. However, first-principles calculations indicated that the formation energy for replacing S atoms with impurity atoms is lower than that for replacing W atoms, suggesting that these atoms could preferentially passivate S vacancies. Nevertheless, their experimental results regarding the effects of In incorporation showed a discrepancy between the theoretical calculations and STEM images, as the latter only revealed In atoms in W sites. The researchers proposed that the significant size difference between In and S atoms could explain this contradiction. They also proposed that increasing the doping concentration, the sample electrical behavior evolves from an n-type material (undoped sample) to ambipolar electrical behavior, while a p-type sample was achieved for heavily doped material; however, in this instance, PL quenching was observed.^3^

Recently, Zhang and collaborators prepared WS_2_ monolayers doped with Ga atoms in situ using the CVD method without the aid of salt. Their results indicate that the PL intensity is enhanced in Ga-WS_2_ samples compared to the pristine sample. Simultaneously, XPS measurements reveal the presence of Ga atoms and a redshift in binding energy, which is associated with changes in the Fermi level as it moves toward the valence band maximum (VBM). In the Raman spectra, it was observed that disorder in the samples increased with the influence of Ga atoms. However, ab initio calculations show the presence of both substitutional Ga atoms and Ga atoms adsorbed on the WS_2_ surface.^19^

We report the growth of In-doped WS_2_ monolayers using atmospheric pressure chemical vapor deposition (APCVD), without employing salt as a catalyst, following a new method proposed for synthesizing WS_2_ monolayers. It has been demonstrated that the defect density in WS_2_ grown without salt is lower than in samples grown with salt as a catalyst.^20^ Additionally, previous studies have yielded contradictory results, highlighting the need for a new, complementary approach. Controllable doping of WS_2_ was achieved by adjusting the precursor mass ratio (WO_3_:In_2_O_3_). Photoluminescence (PL) measurements showed a maximum when the precursor ratio ranged between 10:1 and 3:1 (WO_3_:In_2_O_3_). In contrast, samples produced with ratios of 50:1 and 1:1 exhibited only a slight increase in PL compared to pristine samples. X-ray photoelectron spectroscopy (XPS) analyses revealed the chemical environments of the elements. All doped WS_2_ samples exhibited p-type behavior relative to the pristine sample, as indicated by valence band maximum (VBM) measurements showing a shift of the Fermi level toward the valence band. Raman spectroscopy confirmed that the crystalline structure of the WS_2_ monolayers remained intact when grown in the presence of In atoms in the atmosphere.

## 2 Materials and Methods

### 2.1 Sample Preparation

The WS_2_ monolayers doped with In atoms were synthesized by APCVD in a quartz tube reactor using a horizontal furnace with two heating zones under atmospheric pressure by employing argon gas as the carrier gas. Before growth started, argon gas was allowed to pass for 15 min to guarantee an inert and clean environment. The precursors are powders of WO_3_ (99.995% purity, Sigma-Aldrich) and In_2_O_3_ (99.99% purity, Sigma-Aldrich). They were mixed using a mortar. The mass ratios (WO_3_:In_2_O_5_) were named 1:1, 3:1; 10:1, and 50:1, where the concentration of In_2_O_3_ (10 mg) decreases with respect to WO_3_. Another alumina crucible containing 600 mg of S (Sulfur 99.5%, Sigma-Aldrich) powder was placed upstream inside the reactor. No type of catalyst was employed. The precursor mixtures (WO_3_:In_2_O_3_) were placed on an alumina crucible holding the SiO_2_(275 nm)/Si substrate. The substrate was facedown over the precursor mixture and positioned diagonally within the alumina crucible. The synthesis temperature was 850 °C, and the growth time was 7 min. After each growth cycle, the reactor was cooled to room temperature in an argon atmosphere. A detailed description of the synthesis for pristine WS_2_ can be found elsewhere.^21^

### 2.2 Characterization Techniques

Raman measurements and photoluminescence (PL) maps were conducted using a micro-Raman spectrometer equipped with a charge-coupled device detector. The PL maps were obtained utilizing the piezoelectric stage of an atomic force microscope (AFM) (NT-MDT, NTEGRA SPECTRA). Solid-state lasers with wavelengths of 473 nm (2.62 eV) and 532 nm (2.33 eV) were employed. An 1800 l/mm grating was used for high-resolution Raman spectrum acquisition, providing a resolution of 1 or 0.8 cm^–1^ depending on the laser line. For PL spectra, a 150 l/mm grating was utilized, achieving a resolution of 4 meV. The system was equipped with a 100× magnification objective, and an incident laser power of approximately 20 μW was maintained to generate a laser spot area of about 1 μm^2^. The PL map intensity data reported in the text are based on the average data acquired from multiple maps performed on different samples to ensure reproducibility.

The atomic force microscopy (AFM) measurements were performed using the same NT-MDT equipment mentioned above, operating in contact mode with a silicon tip. The cantilever’s spring constant was 0.204 N/m. We used a scanning rate of 0.5 Hz, corresponding to 5 μm/s, perpendicular to the cantilever’s central axis. The topography images were processed with a second-order plane filter to eliminate tilt between the sample and the microscope. Scanning electron microscopy (SEM) images were obtained using a JEOL scanning electron microscope (model JSM-6701F) equipped with an electron field emission gun.

X-ray photoelectron spectroscopy (XPS) measurements were conducted in a surface analysis chamber under ultrahigh vacuum conditions (pressure of approximately 10^–8^ Pa). The equipment used was a SPECS Phoibos 150, with a collection angle of 90° relative to the analyzer and the sample surface. The X-ray source was a monochromatic Al–Kα (approximately 1486.7 eV). To analyze the XPS spectra, CasaXPS software was employed, utilizing the pseudo-Voigt profile (a linear combination of Lorentzian and Gaussian functions, with the analysis labeled as GL(40), where 40 represents the percentage of the Lorentzian function) and the Shirley background line, both of which were essential parameters for fitting the spectra.

## 3 Results and Discussion

### 3.1 Atomic Force Microscopy

The AFM images were obtained in contact mode. Figure 1 shows topographic images of the pristine WS_2_ sample (Figure 1a) and those prepared with different WO_3_:In_2_O_3_ mass ratios, from Figure 1b–1d. The surfaces were uniform, free of aggregates, and showed no indication of damage due to CVD growth. The height profiles of each sample indicate they are monolayers, with thicknesses typical of a TMD monolayer: 0.8 ± 0.2 nm for the pristine sample; 0.9 ± 0.2 nm for the 50:1 mass ratio; 0.9 ± 0.2 nm for the 10:1 mass ratio; 0.8 ± 0.2 nm for the 1:1 mass ratio. The images showing the typical triangular shape suggest that the growth kinetics remained unchanged with the presence of In atoms in the precursor atmosphere. Scanning electron microscopy (SEM) images in Figure S1 in the Supporting Information (SI) confirm this. SEM images obtained from samples prepared using WO_3_ and In_2_O_3_ oxide mixtures clearly display triangular WS_2_ monolayers as the dominant structures on top of the substrate, with a broad size distribution.

**Figure 1:**
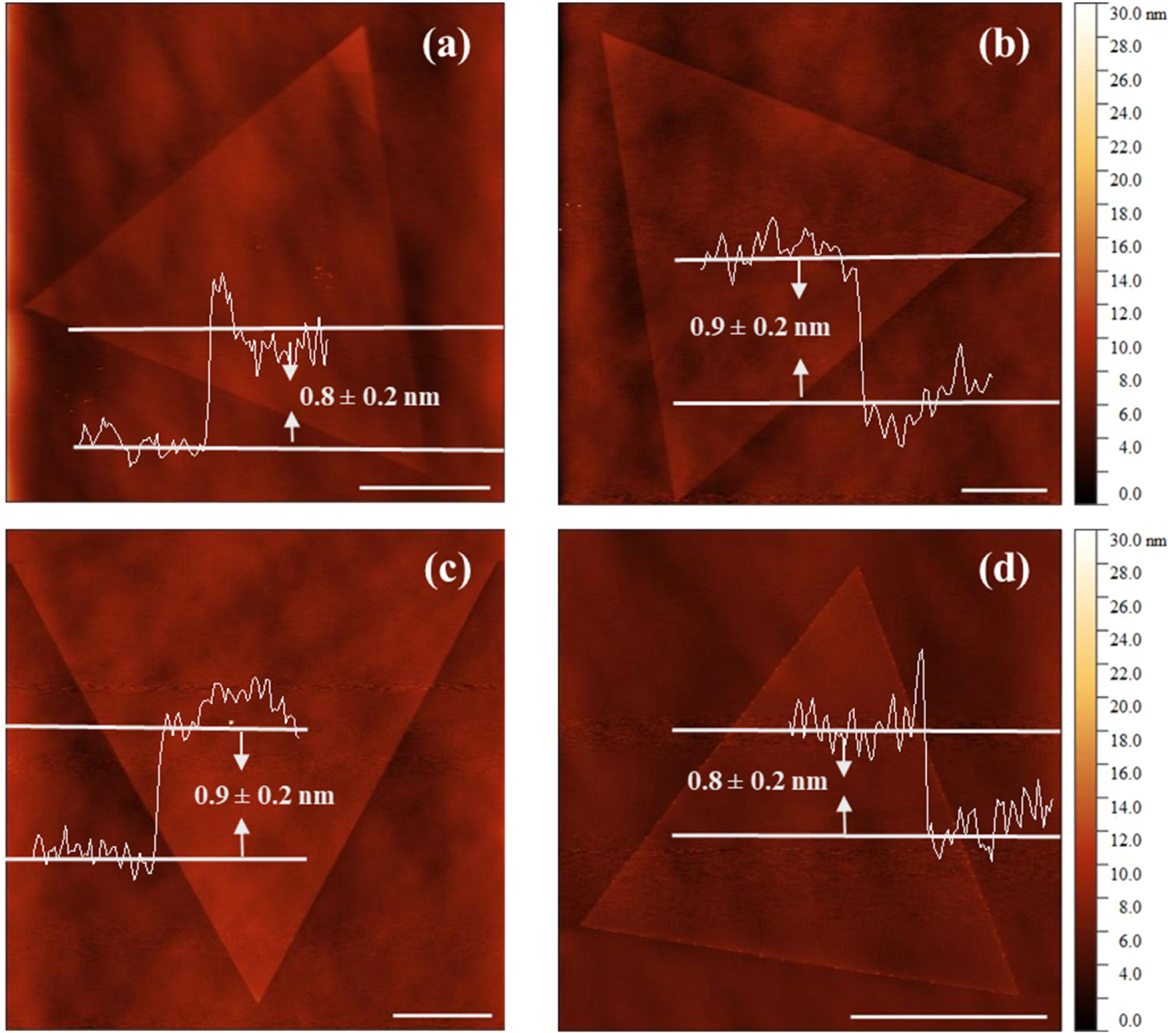
Topographic images obtained by atomic force microscopy (AFM) of the pristine WS_2_ sample (a) and those prepared with different WO_3_:In_2_O_3_ mass ratios: (b–d) images of In-doped WS_2_ monolayers at 50:1, 10:1, and 1:1 WO_3_:In_2_O_3_ mass ratios, respectively. The white scale bar at the bottom of all figures indicates 10 μm. A typical step profile is shown in each image, along with its corresponding value.

### 3.2 Photoluminescence Spectroscopy

The top row of Figure 2a–e displays photoluminescence intensity maps of monolayers from both the pristine sample and samples grown with different WO_3_:In_2_O_3_ mass ratios. On the right side, an intensity scale bar indicates the counts detected by the Charge-Coupled Device (CCD). The top labels correspond to each sample based on its mass ratio. The presence of In atoms during growth results in higher and more uniform PL intensity compared to the pristine sample, especially at mass ratios of 10:1 and 3:1. Conversely, mass ratios of 50:1 and 1:1 show lower luminescence, and the samples are relatively homogeneous. The WS_2_ monolayer images in the middle row (Figure 2f–j) correspond to the full width at half-maximum (FWHM) of the photoluminescence (PL) peak for all samples. The pristine sample and the one with a 1:1 mass ratio display similar FWHM values, while the samples with mass ratios of 10:1 and 3:1 show slightly smaller widths, suggesting a predominance of neutral excitons. The map of the central PL peak position is shown in the bottom row (Figure 2k–o) for all samples. It is evident that the PL peak was not dispersive, indicating uniform PL.

**Figure 2:**
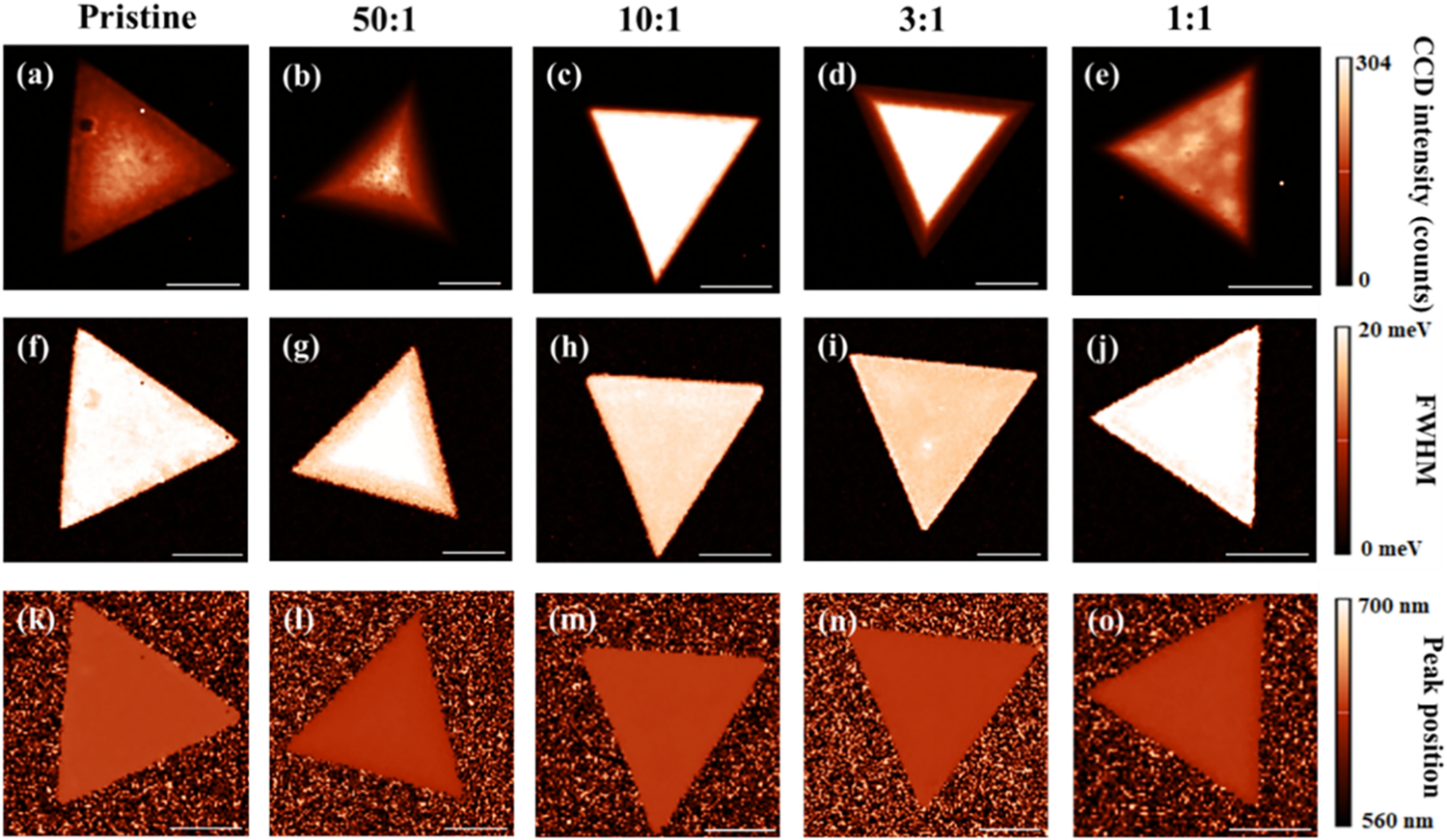
Relevant information for all WS_2_ samples regarding photoluminescence (PL): (a–e) PL intensity; (f–j) full width at half-maximum (FWHM) of the PL peak; and (k–o) PL peak position maps of pristine samples and WO_3_:In_2_O_3_ mass ratios of 50:1, 10:1, 3:1, and 1:1, respectively. The scale bar at the bottom of all figures indicates 10 μm. The maps were obtained using a laser line of 532 nm.

Figure 3a shows the evolution of the photoluminescence (PL) intensity from samples prepared with different WO_3_:In_2_O_3_ mass ratios. The PL intensity data are averaged from several maps obtained from different samples, each prepared with the same precursor mixture, rather than from a single peak intensity of a single monolayer triangle. Additionally, the Si peak was used as a normalization parameter for the PL peaks and maintained at a fixed value during measurements. The pristine WS_2_ monolayer exhibits a direct bandgap of 1.94 eV (with a dashed reference line at 639 nm), while the PL peak position of the samples containing In shows a slight blue shift compared to that of pristine WS_2_. An increase in PL intensity was observed for all samples prepared with In oxide in the precursor mixture, reaching a maximum at mass ratios of 3:1 and 10:1 (W_2_O_3_:In_2_O_3_). In Figure 3b, the evolution of PL intensity is shown in relation to the concentration of In_2_O_3_ in the precursor mixture. The behavior indicates a smaller increase in PL intensity at a 2% In_2_O_3_ concentration. In contrast, a significant rise occurs at 10 and 30% concentrations, followed by quenching back to the initial value at 50%. The increase in PL intensity was up to four times that of the pristine sample at 30% In_2_O_3_ concentration (with a 3:1 mass ratio). The dashed line serves as a visual guide.

**Figure 3:**
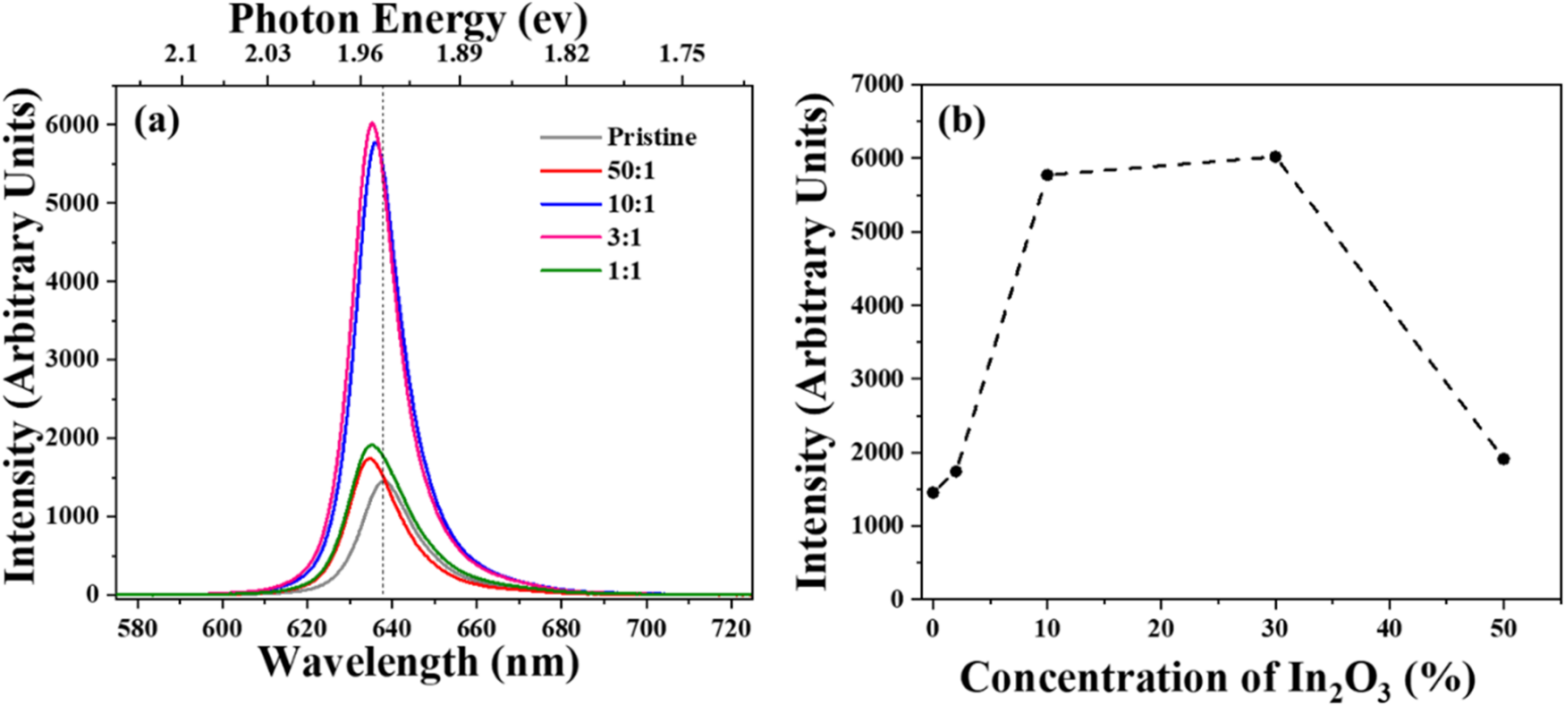
(a) PL intensity evolution with different WO_3_:In_2_O_3_ ratios, and (b) PL intensity behavior with respect to the concentration of In_2_O_3_ (%) in the WO_3_:In_2_O_3_ mixture. All spectra were acquired with a laser line of 532 nm. The dashed line in (a) was placed as a reference at 639 nm (1.94 eV) while the dashed line in (b) serves as a visual guide.

The PL peaks can be deconvolved using two Lorentzian peaks (see Figure 4). The main features dominating the PL emission in WS_2_ monolayers are the excitonic transitions related to the direct bandgap transition at the K point of the first Brillouin zone. These optical transitions are known as X^0^ (ground-state excitons), with other contributions arising from bound states such as trions (X^T^), which consist of two electrons and one hole or one electron and two holes. Trions appear as a small shoulder at lower energies. The main parameters are listed in Table 1.

**Figure 4:**
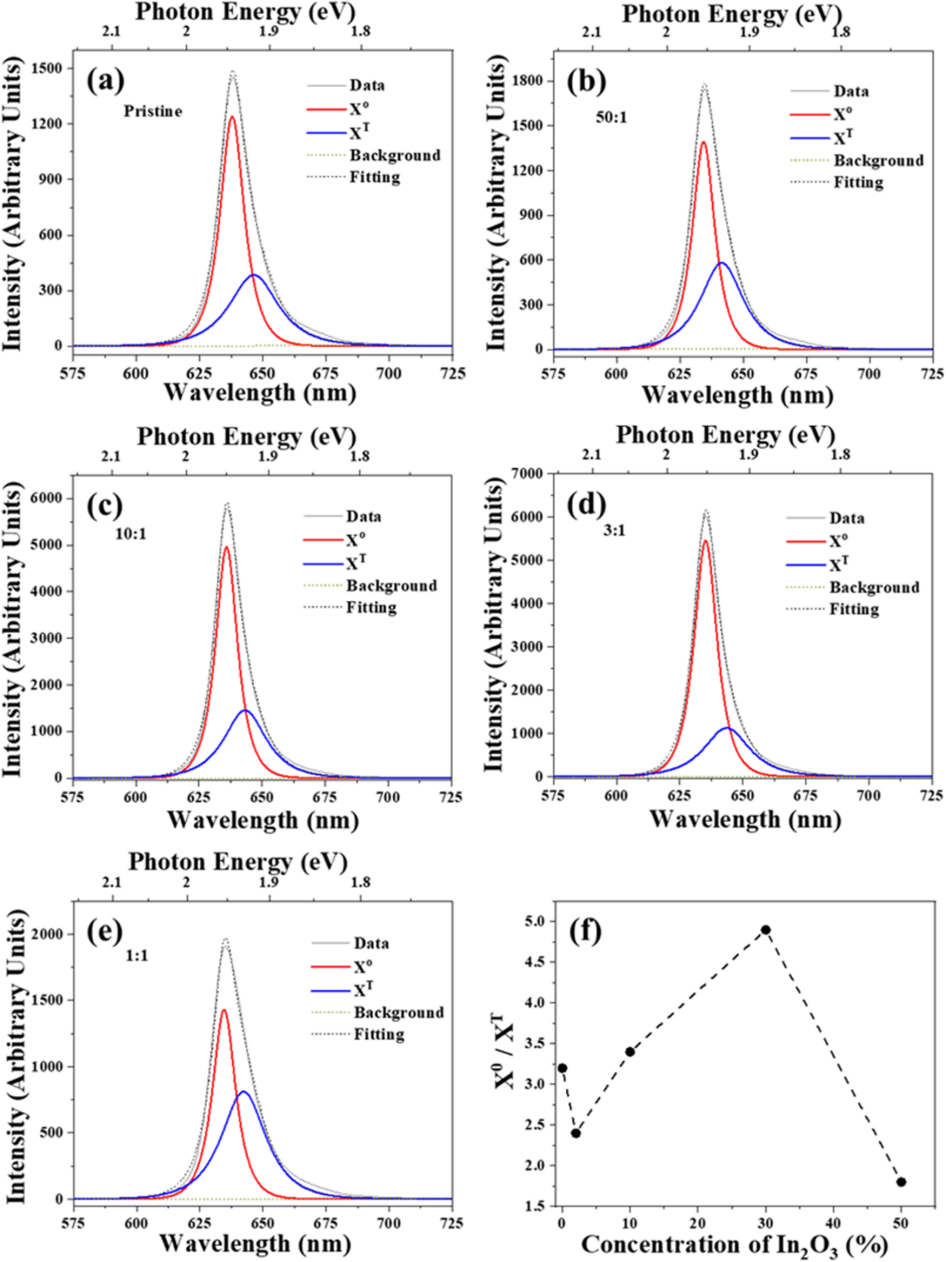
Detailed analysis of the PL peak across all samples, covering the wavelength range from 575 to 725 nm. The central PL peak has been deconvolved into two components for samples prepared with different mass ratios of WO_3_ to In_2_O_3_: (a) pristine; (b) 50:1; (c) 10:1; (d) 3:1; (e) 1:1; and (f) the X^0^/X^T^ ratio as a function of In_2_O_3_ concentration in the WO_3_:In_2_O_3_ precursor mixture. The exciton and trion are represented by the red and blue curves, respectively. All spectra were acquired using a 532 nm laser line.

**Table 1:** PL Intensities (in Arbitrary Units), Peak Positions, and X^0^/X^T^ Intensity Ratio as Functions of the WO_3_:In_2_O_3_ Mass Ratio

WO_3_:In_2_O_3_ ratio	mean intensity	mean position (nm)	X^0^ intensity	X^T^ intensity	X^0^ position (nm)	X^T^ position (nm)	X^0^/X^T^
pristine (WS_2_)	1456	639.0	1240	385	638.0	646.3	3.2
50:1	1743	635.0	1393	580	634.3	641.6	2.4
10:1	5771	636.0	4964	1455	636.0	643.3	3.4
3:1	6022	635.3	5456	1124	635.3	643.6	4.9
1:1	1914	635.0	1432	813	634.7	642.3	1.8

A detailed analysis of PL results was conducted and presented in Figure 4, covering the wavelength range from 575 to 725 nm and including the PL peak of all samples. Figure 4a shows the central PL peak of the pristine sample, which was deconvolved into two components: exciton and trion, represented by the red and blue curves, respectively. The analyses of the doped samples prepared with WO_3_:In_2_O_3_ mass ratios of 50:1, 10:1, 3:1, and 1:1 were displayed in Figure 4b,4c,4d,e, respectively. Comparing these figures, it is evident that the mass ratio of 50:1 exhibits a trion contribution higher than the pristine sample. In contrast, the mass ratios of 10:1 and 3:1 show a trion contribution that is lower than the pristine sample. This trend is shown in Figure 4f, that depicts the X^0^/X^T^ ratio as a function of the concentration of In_2_O_3_ in the precursor mixture. The samples with the highest emission intensity correspond to the highest X^0^/X^T^ ratios, specifically the 10% and 30% concentrations of In_2_O_3_, while the samples with the lowest emission are those prepared with the 2 and 50% of In_2_O_3_ concentrations, respectively.

Table 1 indicates a blue shift of approximately 16 meV in the PL peak for all samples prepared with indium oxide as one of the precursors. This shift suggests that the presence of In atoms in the WS_2_ structure dopes the material and may also be responsible for increasing the contribution of the X^0^ exciton line and the PL intensity. The samples with the highest emission intensity correspond to the highest X^0^/X^T^ ratio, as shown in Figure 4f. This is consistent with a sulfur vacancy passivation process, since these vacancies contribute to PL emission via trion emission. In fact, the contribution of trions to the PL line shape is lower for the sample grown with a mass ratio (WO_3_:In_2_O_3_) of 3:1. At the lowest In concentration, sulfur passivation did not immediately contribute to nonradiative processes; however, in highly doped samples, the incorporation of In introduces new defect states, increasing the number of sites where nonradiative recombination can occur. This recombination enhances the Auger recombination process, which is unrelated to trions or neutral excitons.^22,23^ This effect may result from alloying in the monolayer structures or strain caused by enhanced lattice deformation in the presence of In impurities.

### 3.3 Raman Spectroscopy

Raman spectroscopy is frequently utilized as a standard method for assessing the crystalline quality of single-layer TMDs, particularly WS_2_.^23,24^ Figure 5 presents the Raman spectra obtained from pristine and indium-doped WS_2_ samples using a 532 nm excitation laser. The main features of the Raman spectra across all samples showed no significant shifts in position or broadening. The spectra were unaffected by heat or damage induced during measurements, as their reproducibility was confirmed at different laser powers without any changes.

**Figure 5:**
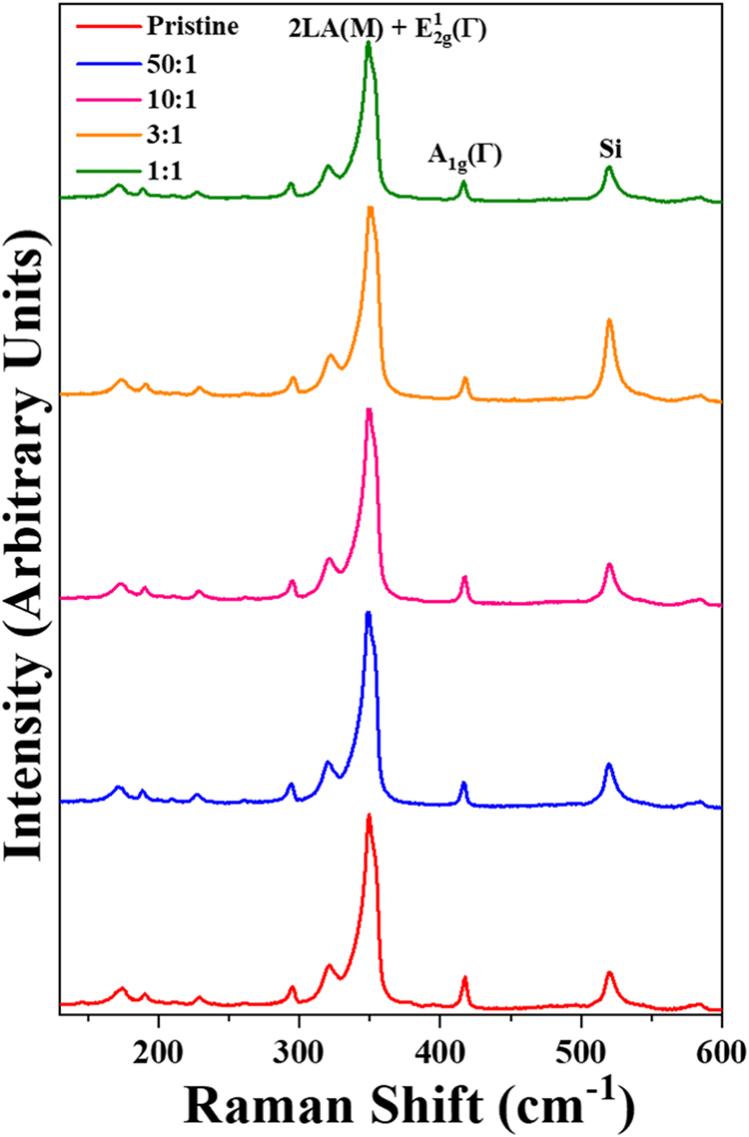
Raman spectra using an excitation laser line of 532 nm. The different WO_3_:In_2_O_3_ mass ratios are indicated in the inset, including the spectrum taken from a pristine sample.

The Raman spectra of the WS_2_ crystal display two main peak contributions: the in-plane 2LA­(M) + E_2g_ mode at approximately 355 cm^–1^ and the out-of-plane A_1_g mode at approximately 419 cm^–1^. Notably, the second-order 2LA­(M), convolved with the E_2_g mode, is the most intense feature in the spectra. The A_1g_ mode is expected to be sensitive to doping effects; however, the peak position change was within the spectrometer’s resolution of 0.8 cm^–1^. Additionally, measurements showed no shift in the E_2g_ mode peak, indicating that the samples were not significantly affected by strain effects due to In incorporation.^25^ The Si peak at 520 cm^–1^ remained unchanged and was used as a reference for the Raman shift. The spectra suggest that indium incorporation occurred without any visible deterioration in the crystallinity of the doped samples. To interpret these Raman results accurately, caution is advised as the information is obtained from deconvolved spectra, and changes in mode positions could be mere artifacts rather than actual changes in the modes. Moreover, the doped samples are compared with the pristine sample, our reference point, which is not defect-free, as claimed in the introduction regarding sulfur vacancies. Thus, our Raman results are consistent with those of the reference,^3^ which also reports no changes in the mode positions. Details of the Raman spectra deconvolution are provided in Table S1 and Figure S2. Nonresonant Raman measurements conducted with a 473 nm excitation laser line produce spectra similar to those discussed above and are shown in Figure S3, with the mode positions listed in Table S2.

Using a laser with an excitation wavelength of 532 nm enables the observation of more features due to the resonance Raman effect. This effect occurs when the excitation laser energy resonantly interacts with the X^b^ exciton of WS_2_, which exhibits an absorption peak at approximately 520 nm (∼2.4 eV).^26^ Specifically, disorder-related modes appear in the lower-frequency part of the spectrum,^27^ and these modes can be used to infer the influence of impurities in the lattice, such as heteroatoms, or to detect changes in defect density compared to pristine samples. In Figure 6a,b, we present the low-frequency Raman spectra obtained from a pristine sample and a monolayer prepared with a WO_3_:In_2_O_3_ mass ratio of 3:1, respectively.

**Figure 6:**
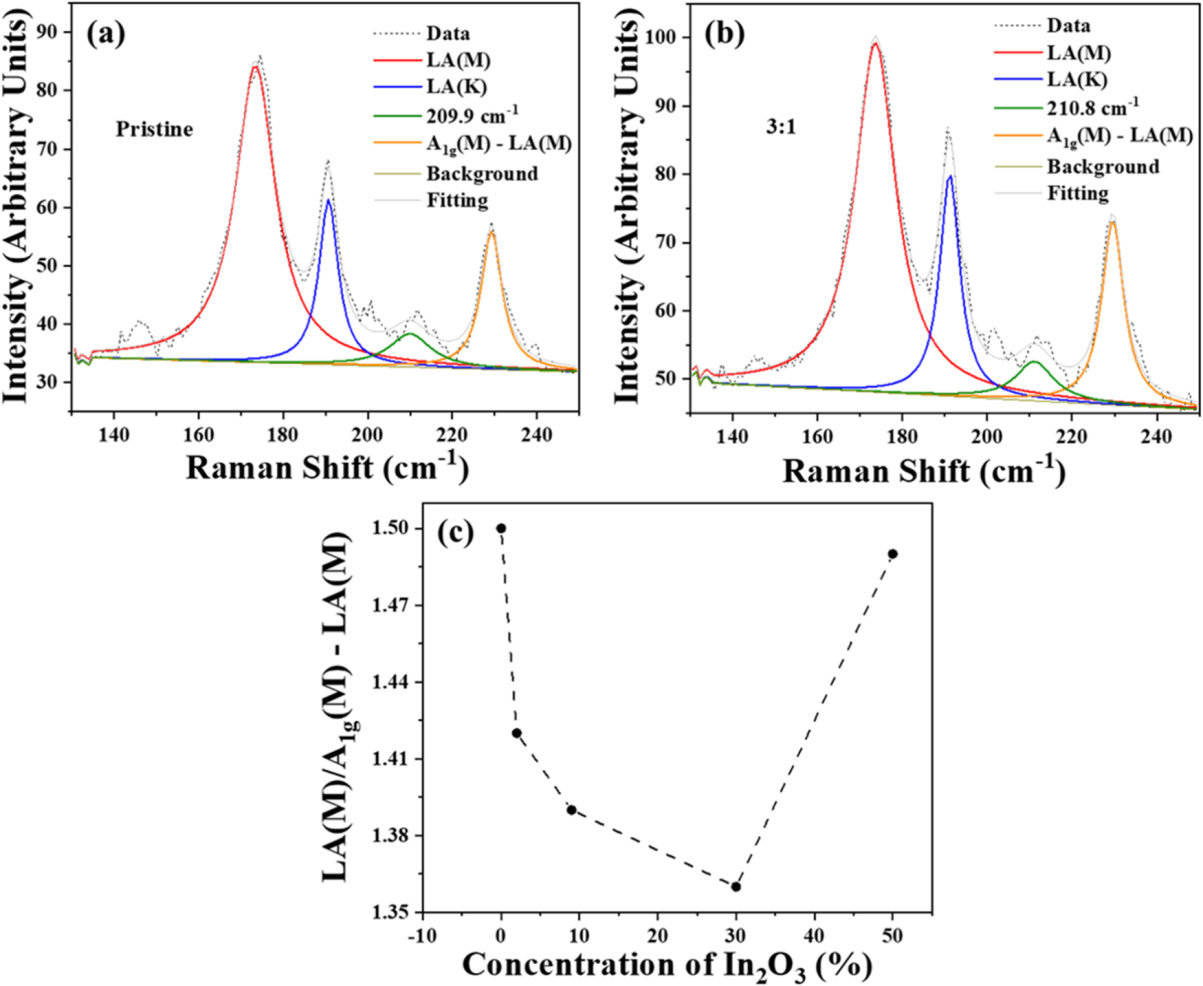
Low-frequency region of Raman spectra obtained using a 532 nm excitation laser line. (a) Pristine sample; (b) sample with a WO_3_:In_2_O_3_ mass ratio of 3:1; (c) LA­(M)/[A1g – LA­(M)] ratio as a function of the In concentration in the WO_3_:In_2_O_3_ mixture. The main features of the spectra are indicated in the inset.

As shown in Figure 6c, the intensity ratio LA­(M)/[A_1g_(M)-LA­(M)] remains practically constant, considering an error of approximately 10%. This ratio LA­(M)/[A_1g_(M)-LA­(M)] is important to qualitatively evaluate the variations in defect density,^26,27^ since the intensity of the LA­(M) mode remains almost constant in all samples, as shown in Table S2. Given that heteroatoms induce disorder effects; this result also indicates that the presence of In atoms in the crystal lattice plays only a marginal role. In Table S2, we list the positions of the main features observed in the low-frequency Raman spectra.

### 3.4 XPS Results

To ascertain the presence of In atoms in WS_2_ monolayers, the XPS technique was employed. It is a highly sensitive method for surface analysis of samples. However, since the X-ray beam has dimensions of approximately 0.5 × 0.5 cm^2^, the values obtained represent an average across many WS_2_ crystals, including contaminants forming clusters on top of some structures as revealed by SEM images (Figure S1).

In Figure 7, we present the XPS survey spectra of samples prepared with different WO_3_:In_2_O_3_ mass ratios—specifically 1:1, 10:1, and pristine—corresponding to Figure 7a,7b,7c, respectively. The main contribution to the spectra originates from the substrate made by silicon oxide: Si2p, Si2s, and O1s. Additionally, a carbon (C1s) signal is observed, attributed to adventitious carbon adsorbed on the sample surfaces during exposure to the atmosphere while transferring from the CVD reactor to the XPS chamber. Tungsten (W4p, W4d, and W4f) and sulfur (S2p) peaks are consistently observed, whereas indium (In3d) is only detected in samples prepared with WO_3_:In_2_O_3_ mixtures as precursors. Each spectrum clearly displays the characteristic peaks of these elements.

**Figure 7:**
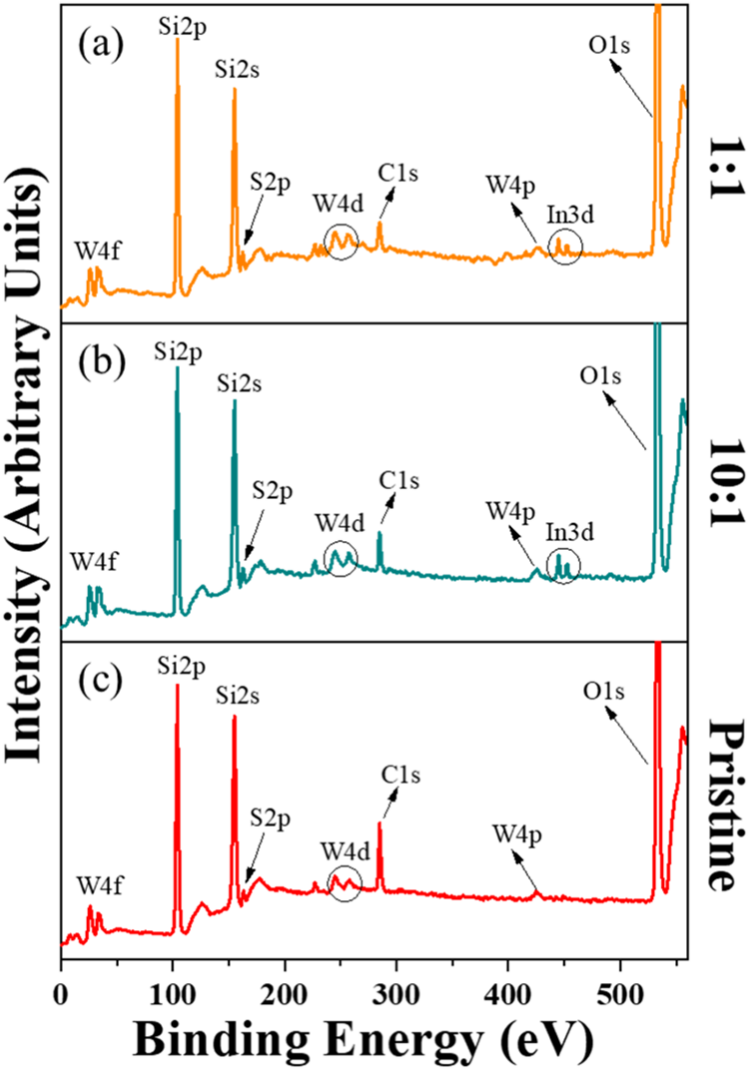
XPS survey spectra of samples prepared with different WO_3_:In2O_3_ mass ratios—specifically 1:1, 10:1, and pristine—corresponding to panels (a), (b), and (c), respectively. All spectra were obtained using an Al monochromatic X-ray source. The elements present in the samples are indicated in each spectrum.

High-resolution XPS spectra of the pristine WS_2_ sample and samples prepared with different WO_3_:In_2_O_3_ mass ratios are presented in Figure 8. The figure includes two panels: one corresponding to the S2p region and the other to the W4f region. In the pristine sample shown in Figure 8a, the doublets S2p_3/2_ and S2p_1/2_ are observed at 162.4 and 163.6 eV, consistent with the oxidation state of S^2–^. Meanwhile, in Figure 8d, the doublets at 32.9 and 35.0 eV correspond to W4f_7/2_ and W4f_5/2_, respectively, which are consistent with the oxidation state of W^4+^. These features are characteristic of WS_2_, with the red lines representing them. Additionally, a small contribution from tungsten atoms in the W^6+^ oxidation state, associated with WO_3_ (W4f_7/2_ at 36.0 eV and W4f_5/2_ at 38.2 eV, indicated by the green lines), was observed in the pristine sample. For doped samples prepared with different WO_3_:In_2_O_3_ mass ratios of 10:1 and 1:1, the XPS spectra are shown in Figure 8b,c, respectively. In these spectra, two doublets appear in the S2p region—one corresponding to WS_2_, similar to that in the pristine sample, and the other doublet is related to the presence of In atoms into WS_2_. Figure 8e,8f shows three doublets in the W4f region: one corresponding to WS_2_, another associated with WO_3_, and an additional one related to the incorporation of In atoms into WS_2_. The feature with the lowest binding energy, indicating the incorporation of In atoms, is represented by the blue lines. Furthermore, the tungsten region exhibits a loss component (W5p) associated with each contribution.

**Figure 8:**
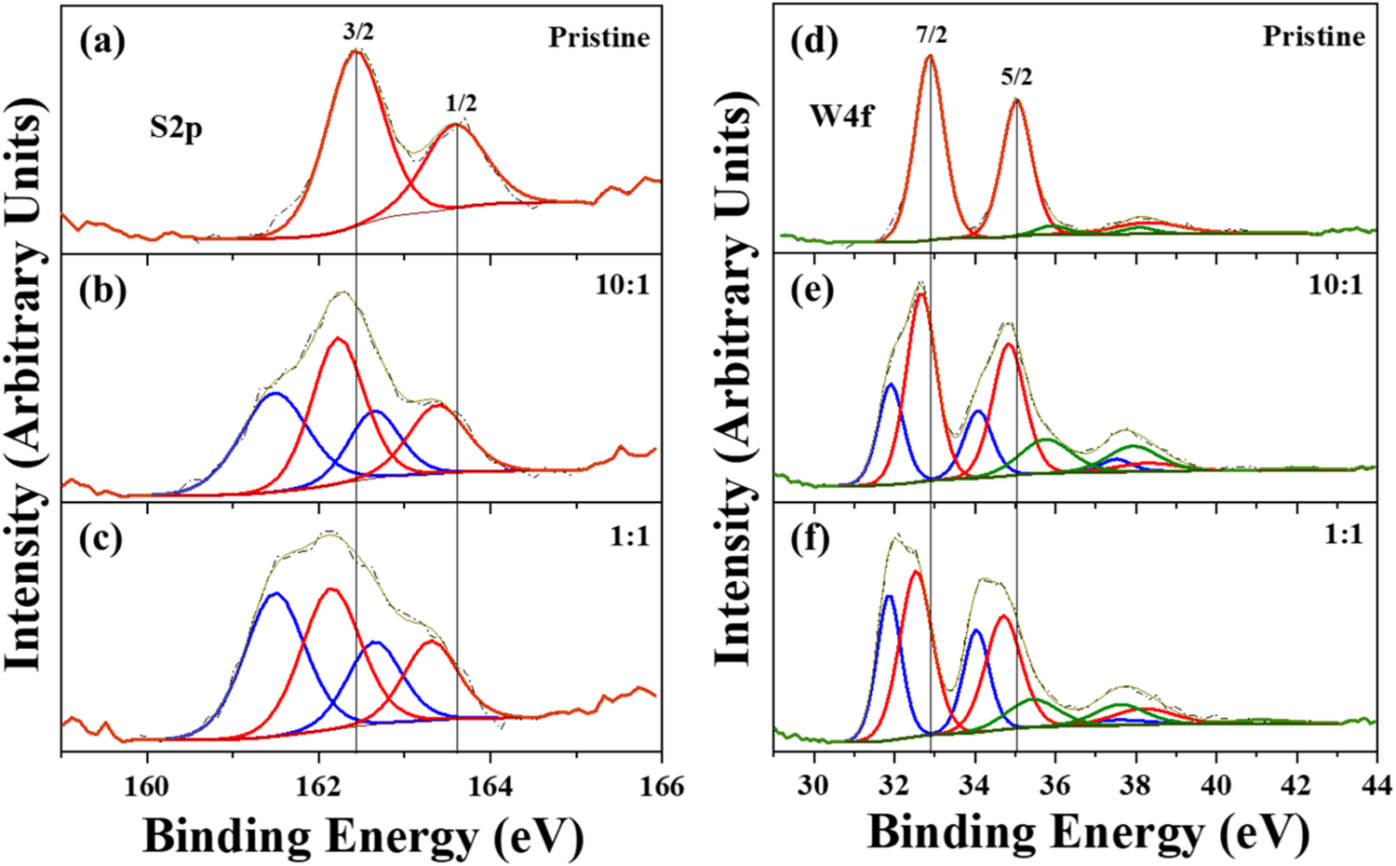
High-resolution XPS spectra of the pristine WS_2_ sample and samples prepared with different WO_3_:In_2_O_3_ mass ratios are presented. The figure includes two panels: one corresponding to the S2p region and the other to the W4f region. (a–c) shows the S2p peak, while (d–f) depicts the W4f peak. All spectra were deconvolved for pristine sample and samples prepared with different WO_3_:In_2_O_3_ mass ratios.

This analysis of the spectra reveals that samples prepared with In_2_O_3_ as one of the precursors indicate that, alongside WS_2_, there is another chemical environment in addition to tungsten oxide. In the left panel, which corresponds to the S2p region, there are two additional peaks at 161.5 and 162.6 eV (shown by the blue lines), consistent with the values reported for ß-In_2_S_3_.^28^ It can be speculated that some clusters on the substrate surface, as shown in Figure 1S, may be responsible for the presence of indium sulfide in the XPS spectrum (Figure 8b,8c). Meanwhile, in the right panel, in addition to the doublets caused by WS_2_ and WO_3_, a new doublet appears in the spectra only when a mixture of oxides was used as a precursor, which is associated with the presence of In atoms. The In–W chemical bond could be responsible for the doublet observed at 31.9 and 34.1 eV (shown by the blue line).

For samples prepared in the presence of In atoms in the atmosphere, the contributions from W^4+^ and S^2–^ states experienced shifts to lower binding energies of 0.24 and 0.26 eV, respectively, compared to the pristine sample. It is also important to note that there is no shift in the position of the WO_3_ peaks, indicating that the oxide was not incorporated into the samples. These changes suggest that the Fermi level shifts toward the valence band in the doped samples, indicating p-type doping.^19,28^

The XPS spectra in the region of In 3d5/2 and In 3d3/2 are shown in Figure S4. They display two peaks at 444.7 and 452.2 eV, which can be attributed to the In^3+^ oxidation state. These positions exhibit slight shifts to lower binding energies—by 0.22 and 0.12 eV, respectively—compared to reported values for bidimensional In_2_S_3_. This suggests that this doublet is due In atoms bonded to W, but the contribution from In_2_O_3_ ^29^ cannot be ruled out.

The valence band maximum (VBM) of the samples can be determined using XPS with a calibrated energy scale, as shown in Figure 9. The figure displays the VBM positions for both pristine and doped samples. Figure 9a corresponds to the pristine sample, where the Fermi edge is modeled using an error function that can also be convoluted by another function, which adds the peak broadening due to temperature. Using it, it was possible to determine the correct fitting (red line) between −2.3 and 2.3 eV, where the Fermi level was 1.72 eV at room temperature. This value is consistent with expectations for the pristine sample prepared by CVD, due to the presence of S vacancies (an n-type behavior). For the doped samples, Figure 9b shows a Fermi level of 1.26 eV for a 10:1 mass ratio, while Figure 9c shows a Fermi level of 1.02 eV for a 1:1 mass ratio. It is evident that, when indium atoms are incorporated into WS_2_, the Fermi level position shifts toward the valence band, moving away from the Fermi level of the pristine sample. This shift indicates p-type doping in all investigated samples prepared with indium oxide as one of the precursors.

**Figure 9:**
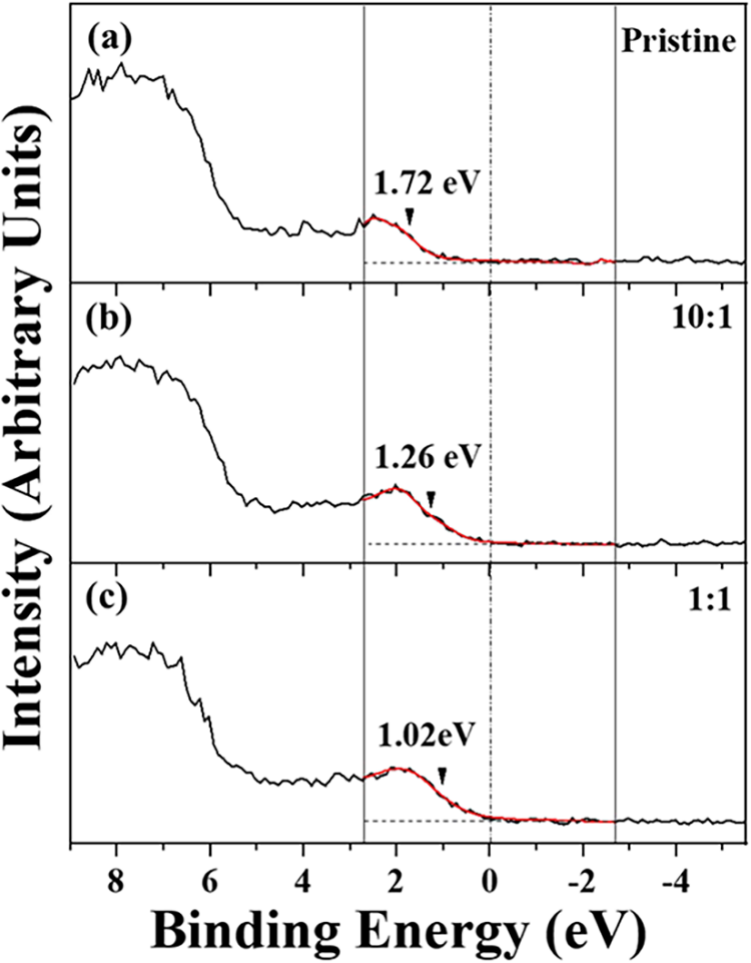
Valence band maximum (VBM) spectra and Fermi level positions obtained by XPS. (a) For pristine WS_2_, the Fermi level position was ∼1.72 eV, while for samples prepared with different WO_3_:In_2_O_3_ mass ratios, there is a clear shift in the Fermi level position. (b) and (c) show the values of ∼1.26 and 1.02 eV corresponding to 10:1 and 1:1 mass ratio, respectively.

## 4 Summary and Conclusions

AFM and PL measurements confirm the monolayer character of the analyzed triangles, whose surface was uniform and free of aggregates, whereas SEM images clearly show that the samples prepared using a mixture of WO_3_ and In_2_O_3_ as precursors were essentially WS_2_ monolayers (triangular structures) with a broad size distribution. As is clear from SEM images, each sample was composed of a combination of hexagonal WS_2_ monolayers and some clusters, probably In-rich, the former being the dominant one.

In the experimental setup used, the laser beam spot size was approximately one square micrometer. Since WS_2_ monolayers exhibit a direct bandgap transition, they can be easily distinguished from other structures, such as multilayers, due to their significantly higher luminescence intensity. Consequently, the Raman and PL results presented in Section 3 were certainly obtained from monolayer crystalline structures. In fact, Raman results revealed that the crystalline structure was preserved in samples prepared with In in the precursor atmosphere. The PL spectra of samples prepared with In displayed a slight blue shift compared to the pristine sample, indicating p-doping. An analysis of the XPS data further indicates that the CVD synthesis of WS_2_ using a WO_3_:In_2_O_3_ oxide mixture as a precursor produces p-doped samples. The observed shift in the W and S peaks, corresponding to the W^4+^ and S^2–^ oxidation states, respectively, suggests a change in the Fermi level toward the valence band, strongly implying p-type doping in WS_2_. The shift of the VBM position shown in Figure 9 supports this interpretation. The combined XPS and PL results demonstrate that all In-doped samples exhibit p-doping behavior. First-principles calculations have shown that the formation energy for replacing S atoms with In atoms is lower than that for replacing W atoms.^3^ Furthermore, a recent publication has shown that another group IIIA atom, Ga, can also be preferentially adsorbed on top of an S position, generating gap states near the top of the valence band and resulting in p-doped material, consistent with our results.^19^ Compared to previous investigations on In-doped WS_2_,^3–11^ our results consistently agree with each other and with first-principles calculation.

In addition to p-doping, the incorporation of In atoms results in up to a 4-fold increase in PL intensity. These results may be caused by the replacement of S vacancies by In atoms at S sites within the material structure, as we observe that maximum emission intensity occurs when there is an increase in neutral exciton recombination. That is, the incorporation of impurities stabilizes the intrinsic defects to such an extent that they become the dominant defect type.

In conclusion, our work demonstrated an in situ doping process to incorporate In atoms into WS_2_ monolayers. The most significant effects are, on the one hand, an increase in the PL emission intensity of up to four times compared to pure WS_2_ monolayers, and on the other hand, p-type doping of the samples. These results provide a foundation for refining the controlled preparation of In-doped WS_2_ by CVD, enhancing its potential for optoelectronic applications.

## Supplementary Material



## Notes

SEM images taken from different sample regions prepared with a 1:1 mass ratio of WO_3_ to In_2_O_3_ (Figure S1); deconvolution of spectra obtained from (a) pristine sample; (b) 50:1; (c) 10:1; (d) 3:1; (e) 1:1 sample prepared with different oxide mixtures of WO_3_ and In_2_O_3_ (Figure S2); Raman spectra were obtained with a 473 nm excitation laser line (Figure S3); position of peaks present in Raman spectra shown in Figure S2 obtained using a laser with a wavelength of 532 nm (Table S1); position of peaks present in Raman spectra shown in Figure S3 obtained using a laser with a wavelength of 473 nm (Table S2); XPS spectra of In region for various WO_3_:In_2_O_3_ mass ratios: (a) 10:1; (b) 1:1 (Figure S4) (PDF)
