# Supplementary material for: Indium Incorporation into Tungsten Disulfide Monolayers during Chemical Vapor Deposition Growth: Doping and Enhancement of Photoluminescence
Source: ACS Omega. 2025 Sep 15;10(38):44280–90. doi: 10.1021/acsomega.5c05880 (PMC12489619; doi:10.1021/acsomega.5c05880)
Supplement: Supplementary file 1 [file ao5c05880_si_001.pdf]

Indium incorporation into tungsten disulfide monolayers during chemical vapor deposition growth: doping and enhancement of photoluminescence.

Neileth Stand[1]*, Cesar D. Mendoza[2], Fernando Lázaro Freire Jr.[1]

[1]Pontifícia Universidade Católica do Rio de Janeiro, Departamento de Física. Rua Marquês de São Vicente, 225. Rio de Janeiro, 22451-900, Brazil.

[2] Universidade do Estado do Rio de Janeiro, Departamento de Engenharia Elétrica, Rua São Francisco Xavier, 524, Rio de Janeiro 20550-900, Brazil.

*Corresponding author e-mail: nstandfigueroa@vdg.fis.puc-rio.br

1. SEM images

Sample prepared with a mass ratio 1:1 (WO_3:In_2O_3)

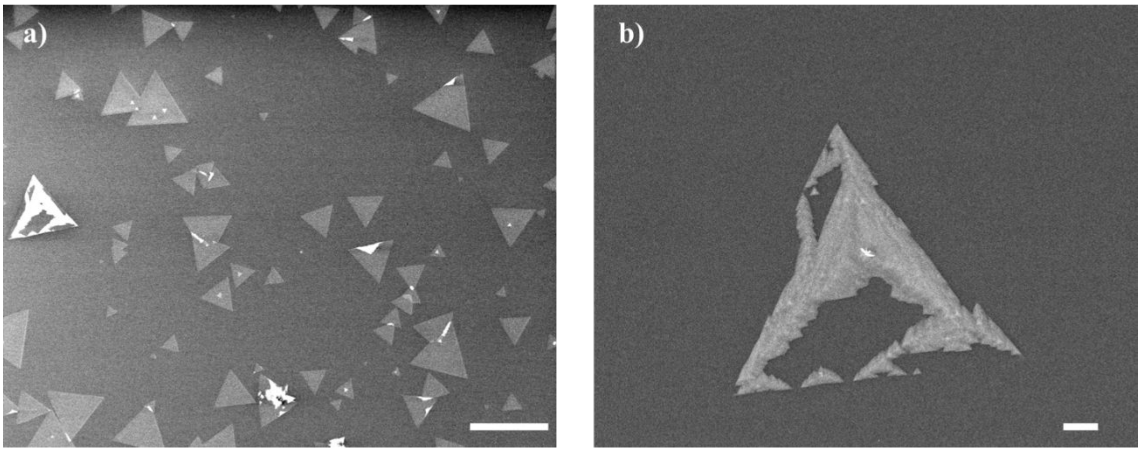

Figure S1. SEM images taken from different sample regions prepared with a 1:1 mass ratio of WO_3 to In_2O_3. The white bars represent the scale, with 100 µm in (a) and 100µm in (b).

The area is primarily covered by typical triangles of WS_2 monolayers with a broad size distribution. SEM images revealed the presence of several different structures in the sample, suggesting multilayer formations or clusters, while the characteristic hexagonal structure of WS_2 monolayers dominates the images.

2. Raman Spectroscopy

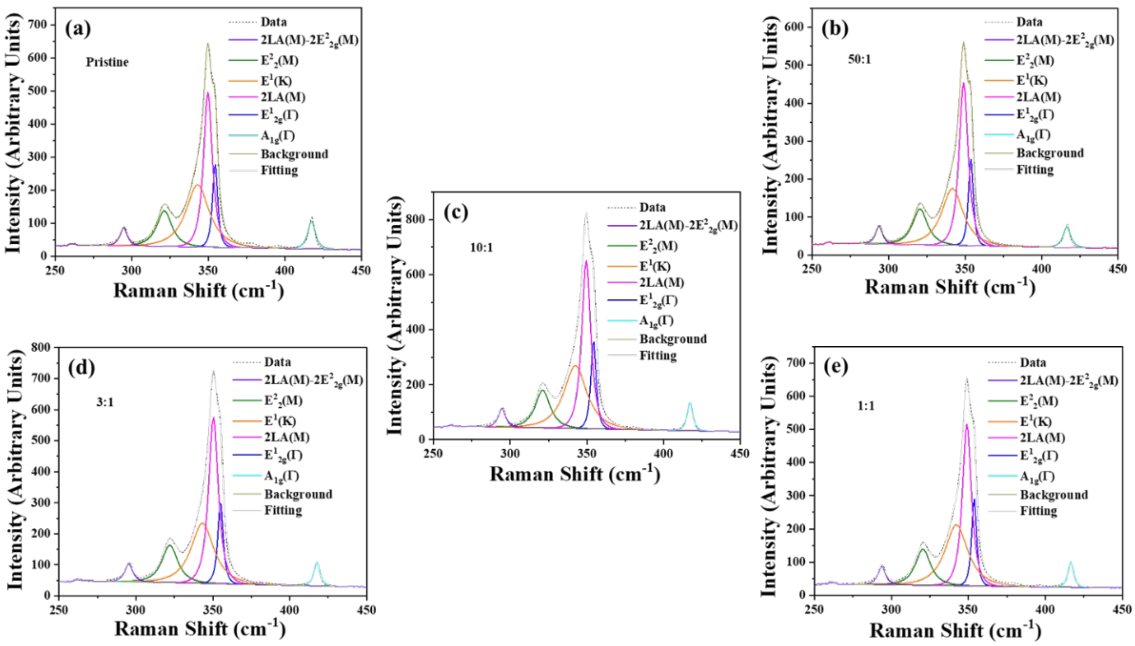

Figure S2. Deconvolution of spectra obtained from (a) pristine sample; (b) 50:1; (c) 10:1; (d)3:1; (e) 1:1 sample prepared with different oxide mixtures of WO_3 and In_2O_3. The laser wavelength was 532 nm.

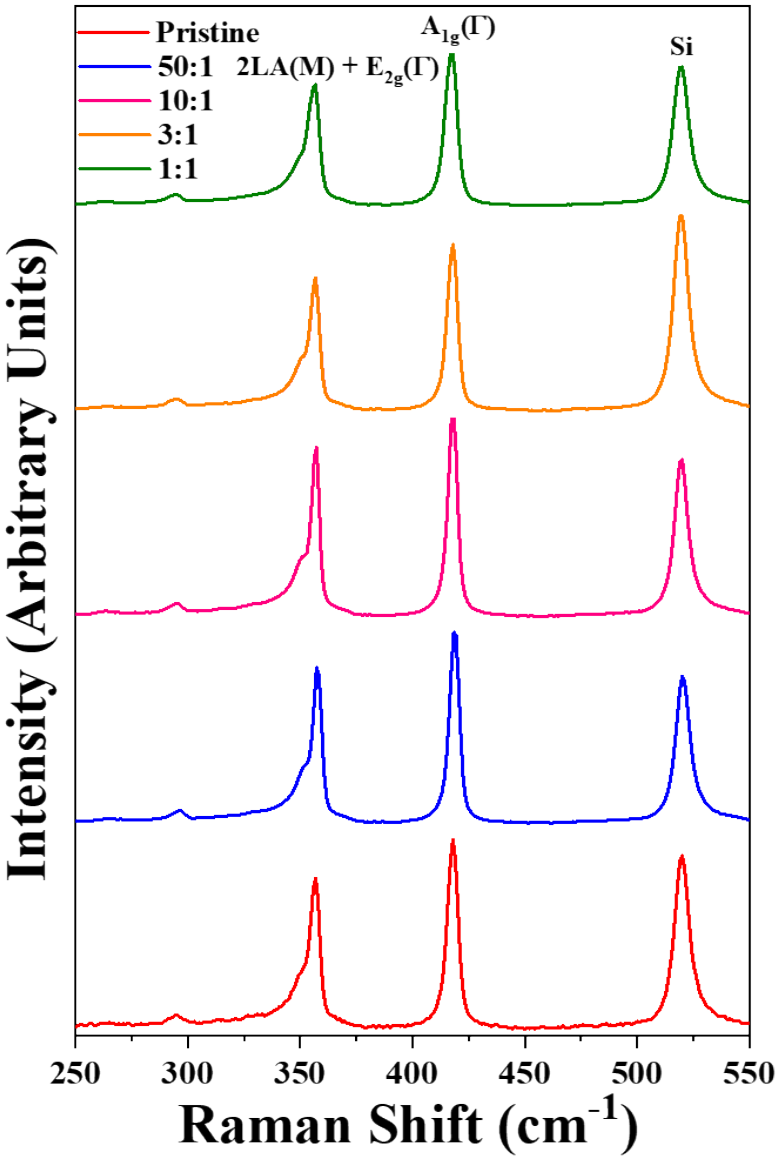

Figure S3. Raman spectra were obtained with a 473 nm excitation laser line. The different WO_3:In_2O_3 mass ratios are shown in the insert, along with the spectrum from a pristine sample.

Table S1: Position of peaks present in Raman spectra shown in Figure S2 obtained using a laser with a wavelength of 532 nm. All the information is associated to ± ΔE, in this case is ± 0.8.

WO_3:In_2O_3 Ratio	$A_{1g}(\Gamma)$		$E^1_{2g}(\Gamma)$		2LA(M)		$E^1(K)$		$E^2_2(M)$		2LA(M)-2E^2_{2g}(M)		Normalized 2LA(M) with respect to the deconvoluted area
	Position (cm^{-1})	FWHM	Position (cm^{-1})	FWHM	Position (cm^{-1})	FWHM	Position (cm^{-1})	FWHM	Position (cm^{-1})	FWHM	Position (cm^{-1})	FWHM	
Pristine (WS_2)	416.9	5.9	354.6	4.0	349.6	7.0	343.0	18.3	321.6	11.8	294.2	5.6	0.33
50:1	416.5	5.9	354.0	3.8	349.1	7.2	341.6	17.1	320.9	11.8	293.5	5.4	0.38
10:1	416.9	4.4	354.6	4.0	349.6	7.1	342.2	18.0	320.7	11.6	294.2	5.7	0.36
3:1	417.7	4.7	354.6	4.2	350.5	7.6	343.1	17.9	322.4	12.4	295.1	6.2	0.37
1:1	416.5	4.4	353.9	3.8	349.0	7.0	342.4	17.8	320.8	11.9	293.4	5.9	0.36

Table S2: Position of peaks present in Raman spectra shown in Figure S3 obtained using a laser with a wavelength of 473 nm. All the information is associated to ± ΔE, in this case is ± 1.

WO_3:In_2O_3 Ratio	$A_{1g}(\Gamma)$		$E_{2g}(\Gamma)$		2LA(M)		$E^2_2(M)$		2LA(M)-2$E^2_{2g}(\Gamma)$		2LA(M)-3E^2_{2g}(M)		Normalized 2LA(M) with respect to the deconvoluted area
	Position (cm^{-1})	FWHM	Position (cm^{-1})	FWHM	Position (cm^{-1})	FWHM	Position (cm^{-1})	FWHM	Position (cm^{-1})	FWHM	Position (cm^{-1})	FWHM	
Pristine WS_2	418	5	357	4	350	11	332	35	294	9	265	15	0.18
50:1	418	5	357	4	351	9	335	28	296	9	267	18	0.14
10:1	418	5	357	4	351	10	335	34	295	9	266	14	0.17
3:1	418	5	358	4	350	10	334	34	294	11	266	27	0.16
1:1	417	6	356	5	349	11	331	30	294	10	264	11	0.17

3. XPS

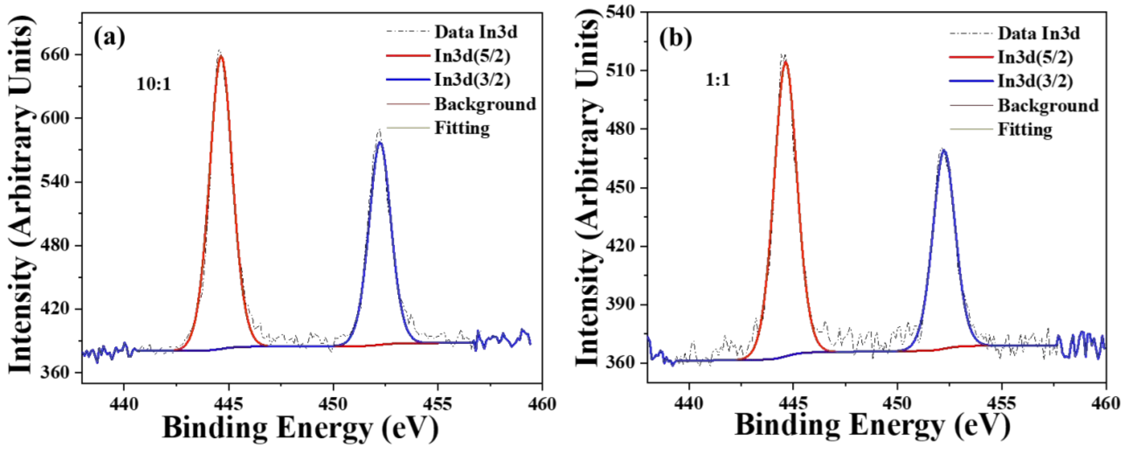

Figure S4. XPS spectra of In region for various WO_3:In_2O_3 mass ratios: (a) 10:1; (b) 1:1.
